# Using energy time–frequency of Hilbert Huang transform to analyze the performance of the variable valve timing engine

**DOI:** 10.1038/s41598-022-06404-3

**Published:** 2022-02-11

**Authors:** Arshed Abdulhamed Mohammed, Sallehuddin Mohamed Haris

**Affiliations:** 1 Department of Materials Engineering College of Engineering, University of Diyala, Baquba, Diyala Iraq; 2 Department of Mechanical and Manufacturing Engineering, Faculty of Engineering and the Built Environment, Universiti Kebangsaan Malaysia, 43600 UKM, Bangi, Selangor Malaysia

**Keywords:** Engineering, Mechanical engineering

## Abstract

In this study, a diagnosis method was successfully implemented to identify different sounds coming from individual mechanical parts within a group of engine moving parts controlled through a variable valve timing system. The novelty of this diagnosis method is in the determination of specific sounds coming from each part within this group when they are in good working condition and without any defects. This will facilitate in early detection of faults occurring on the parts, identified through changes in the sound wave energy. Through this study, this diagnosis method was validated in three ways, namely the consistency of the results with previous studies, the synchronization of sounds from mechanical parts in overlapping cases, and the cross-correlation of engine sound modes that results from analysis using the Hilbert Huang Transform. In this paper, the distribution of sound energy according to its frequencies was utilized to distinguish which of the engine combustion chambers of a Dodge Journey 2.4 was faulty. To conduct that, the noise-based test technique was selected to record the engine sound. The results show that there is a link between the RMS energy of the engine sound and the engine output torque.

## Introduction

Today, cars are regarded as a high energy consumption source, and one of the biggest sources of carbon emissions. The variable valve timing (VVT) system is regarded as one of the most important solutions proposed to overcome these problems. The VVT system has the ability to cut down fuel consumption, control carbon emissions, and at the same time, enhance the performance and capability of gasoline engines^1^. Controlling the opening and closing times of the engine combustion chamber valves were found to be an efficient way to achieve these aims, and the computer-operated solenoid and sensor system performs this role in the VVT system^2^.

In spite of the advantages provided by VVV, it, however, contributes to the increased complexity of the general systems of the car and raises the cost of production. It also imposes increased difficulty in the diagnosis of engine malfunctions, as the VVT system is an intelligent control system with the ability to generate compensation for any reduction in engine power due to engine faults^3^. Such difficulties do not augur well for the well-being of automotive maintenance workers, and studies have found a high prevalence of musculoskeletal disorders among them^4,5^.

In the same context, methods for engine malfunction diagnosis have also been developed. In general, these methods can be divided into four categories. The first is by checking the engine performance^6^. However, this method tends to be effective in cases where the defect has become serious but fails in early-stage detection. The second method is by examining wear particles or filings in the engine oil^7^. Despite the accuracy of this method, it requires expensive equipment and lengthy analysis to obtain results. The third is through vibration tests^8^, and lastly, through noise-based tests^9,10^. The last two methods are similar in that they treat vibration and noise as received signals. They differ from each other in that vibration tests require a direct connection to record the signals, but noise-based tests do not^11^.

Noise-based tests comprise two main approaches, the pattern recognition approach (APR) and the characteristic extraction approach (CEA). APR involves artificial intelligence methods such as neural networks, genetic algorithms, and fuzzy logic. Fukada and Yasuda^12^ utilized a neural network technique to diagnose the fault in the engine through the crankshaft angle data. Wear^13^ used radial basis functions neural networks to detect changes in engine pressure. Verification tests indicated that the method was effective in engine fault diagnosis. Multi-objective optimization and genetic algorithms have also been used to analyze exhaust gases to detect incipient faults of IC engines^14^. Zuo^15^ developed a fuzzy logic-based system for the diagnosis of starter motor faults in emergency vehicles.

CEA techniques, except for FFT, have some advantages not found in APR. One such feature is the ability to analyze and represent the sound energy spectrum in the time–frequency domain, which may be performed using the wavelet transform (WT) or the Hilbert–Huang Transform (HHT). In this study, this feature is used to detect and recognize sounds from the movement of engine parts. The engine sound is a non-stationary type of signal^16^, and CEAs are especially suited to analyze them^17^. WT was used to diagnose faults in an IC engine and its cooling system in^18^. Wrobel and Kazmierczak^19^ monitored engine performance by using Laser Doppler Vibrometry instead of recording data using sensors. They then analyzed these data by utilizing WT. Although the work was successful, the method was deemed expensive. The second technique in CEA is the HHT. In addition to having all CEA characteristics, HHT offers two further advantages over WT. The first is the ability to analyze non-linear signals, which WT is incapable of, and the second is that it is an adaptive technique, in contrast to WT which is a priori technique^20–23^. Engine noise is a nonlinear signal in its nature^24^ this gives preference to HHT over WT, whereas WT needs another software to conduct analysis to this signal such as Volterra filter^25^ or filter from the MIT-BIH database^26^. As a matter of fact, the use of these types of filters causes increasing systematic errors in analyzing the signal.

In recent times, there has been a rise in attention on the use of HHT. This includes many applications such as using HHT in the detection of motor faults^27^, detection of existing fractures in the movable mechanical arms^28^, and detection of distortions in ball bearings^29,30^. Belshein et al.^24^ tried to enhance the empirical mode decomposition in HHT for diagnosis of some faults inside the engine. Most studies using CEA techniques in the detection of engine faults, including previous studies using HHT, are based on comparing faulty engines to the standard case of the unbroken engine.

In this work, our focus was on diagnosing the performance of mechanical parts controlled by the VVT system during engine idling. One feature of HHT is the ability to decompose the signal into several modes which are independent of each other. This feature was used to break up sounds from the engine into several modes, where each mode resulted from the movement of one particular part of the mechanical system controlled by the VVT. The results were then verified in three ways, which are by comparing for close agreement with previous studies, synchronization among the analyzed signals, and the cross-correlation technique. The significance of this study comes from the mapping of unique HHT signal characteristics to particular motions of the engine components in a healthy state, without defects. This information will be helpful in the early identification of engine faults that may occur in the future.

## Hilbert–Huang transform (HHT)

The empirical mode decomposition (EMD) is regarded as the cornerstone of HHT. EMD results in the sifting of a signal into a group of intrinsic mode functions (IMF). Each IMF in this group of IMFs is independent of one another^31,32^. The engine sound is made up of sounds from a group of moving mechanical components. Sifting the engine sound into several independent IMFs means that each IMF may refer to a specific component of the engine. This is what this study tries to prove in the remainder of this paper.

In this following, $I_{k} \left( t \right)$ denotes the $k$-th IMF, where $k = 1,2, \ldots , n$, and $n$ is the number of IMFs.Any signal, such as a noise or sound wave, in the time domain $x\left( t \right)$, can be decomposed into a number of IMFs, and the remainder is denoted as $G_{n} \left( t \right)$ as shown in Eq. (1).

1 $$x(t) = \mathop \sum \limits_{i = 1}^{n} I_{k} (t) + G_{n} (t)$$

The Hilbert transform, $\alpha_{i}$ for each $I_{k} \left( t \right)$ is given by

2 $$\alpha_{i} (t) = \frac{1}{\pi }L\mathop \smallint \limits_{ - \infty }^{\infty } \frac{{I_{k} (\tau )}}{t - \tau }d\tau$$

where $L$ is the Cauchy principal value^33^.Then, denote $M(t)$ as

3 $$M(t) = I_{k} (t) + j \alpha_{i} (t) = V_{i} (t)e^{{j\psi_{j} (t)}}$$

$V_{i} (t)$ represents the energy of each IMF, where

4 $$V_{i} (t) = \sqrt {[I_{k} (t)]^{2} + [\alpha_{i} (t)]^{2} }$$

And $\psi_{i} (t)$ is the conformable phase given by

5 $$\psi_{i} (t) = \tan^{ - 1} [\alpha_{i} (t){/}I_{k} (t)]$$

The instantaneous frequency $\omega_{i} (t)$ is obtained from the derivative of Eq. (5),

6 $$\omega_{i} (t) = (1/2\pi )[d\psi_{i} (t){/}dt]$$

It can be expressed as the distribution of energy in time–frequency, which is the Hilbert spectrum, $H(\omega ,t)$, as

7 $$H(\omega ,t) = Re\mathop \sum \limits_{i = 1}^{n} \omega_{i} (t)e^{{j\smallint \omega_{i} (t)dt}}$$

where $j = \sqrt { - 1}$ is the imaginary number.

The marginal spectrum $h(\omega )$ is one way to express the value of energy for each frequency ^34^. $h(\omega )$ is defined as

8 $$h(\omega ) = \mathop \smallint \limits_{0}^{Q} H(\omega ,t)dt$$

where $Q$ is the total data length.

## Test equipment and setup

**Figure 1:**
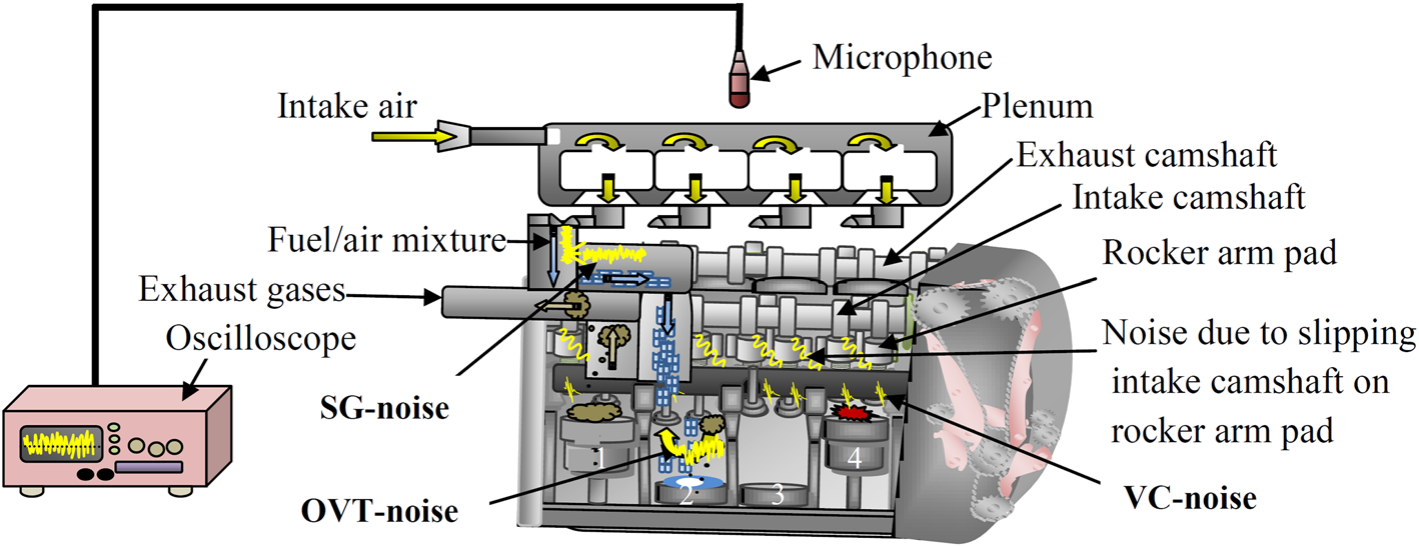
Schematic clarification of Dodge Journey engine based-noise test.

In this research, noise-based tests were conducted to record the engine sound. The dual VVT engine noise of a 2013 Dodge Journey was recorded with a FeiYa dynamic microphone. This is a half-duplex signal transmission microphone. The half-duplex pickup prevents noise interference coming from other directions and amplifies the high gain sound coming from the targeted direction^35^. The microphone was oriented directly towards the engine cover, thus the only movements of the engine components were recorded. Figure 1 illustrates the test setup.

Most component movements under the engine cover are controlled by the VVT system. These include movements of both the intake and exhaust valves, the camshaft, and the flow of air and fuel during the suction stroke and exhaust strokes. Reference^36^ reported that the loudest sounds emitted by moving mechanical parts of the engine come from the group of components controlled by the VVT system. In the tests, a DSEX1102A (100 MHz) oscilloscope was used to display and record sound signals captured by the FeiYa dynamic microphone. It is worth noting that this oscilloscope has a sampling rate of 2 gig samples/second, giving it the ability to record the finest details of the measurement.

## Experiment methodology and discussion

The data acquisition was performed with the oscilloscope set to the high-resolution mode. The data was saved as Excel files, from which analysis was performed using MATLAB. Classifying sounds from the movement of separate undamaged engine parts that are controlled by the VVT system is one of the aims of this study. The data would be used for early detection of any engine component defect in the future.

The tests were carried out with the engine idling, and the engine speed was gradually increased from 1000 to 4000 rpm, in steps of 1000 rpm. Under normal working conditions, the engine stabilizes at speed of 650 rev/min, but an initial speed of 1000 rpm was used to maintain a constant speed increase percentage, and 4000 rpm was the maximum engine speed at idling. In the following, three procedures were conducted to verify the correctness of the diagnoses is described.

### Coincide the experimental results with previous studies

**Figure 2:**
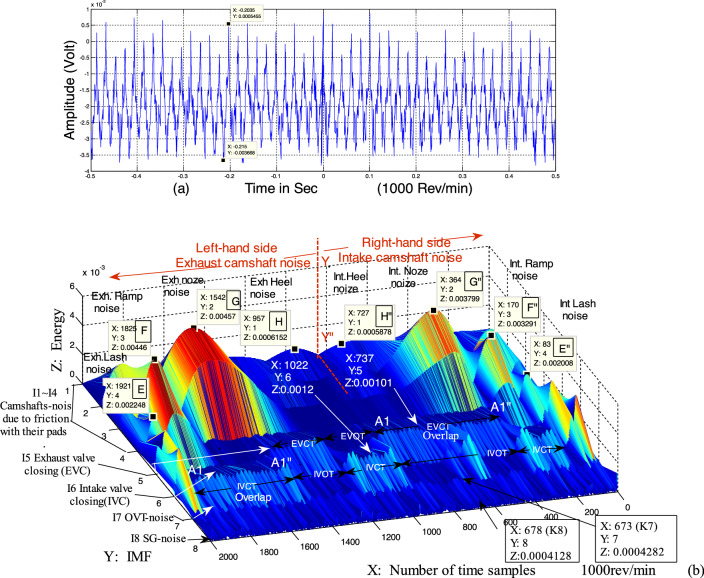
(**a**) Original recorded signal at 1000 rpm and (**b**) 3-Dimensional distribution of energy on the frequency-time plate.

This study follows the general outlines of previous similar studies. However, some details that have not been presented before are highlighted here. Such details can be useful in the early detection of engine faults. Cheng et al.^36^ mentioned that the acoustic pressure wave is emitted with the noise of the engine. These acoustic waves are generated in three ways: (a) during the suction stroke, a negative pressure wave is transmitted from the combustion chamber (CCh) to the plenum of the engine. This transmission causes high noise inside the plenum, as shown in Fig. 1. This noise is called suction-generated noise (SG-noise). (b) During the overlap valve-timing, other negative waves travel from CCh to the plenum. This second negative wave causes a noise called overlap valve-timing noise (OVT-noise)^37^, as shown in Fig. 1c Noise due to successive strikes through the valve closing events (VC-noise), as shown in Fig. 1^38^. The recorded engine signal sound is shown in Fig. 2a. The signal is a complex sinusoidal wave, indicating that it carries a lot of information. It is also a nonlinear and non-stationary signal, making HHT a suitable method of analysis.

Figure 2b represents a 3-dimensional analysis of the original signal (Fig. 2a). The overall view indicates that there are three types of modes according to their frequencies. These modes correspond to the similarity at high frequencies, between I7 and I8, the similarity at medium frequencies, between I5 and I6, and that at the lowest frequencies from I1 to I4.

It is known from previous studies that the flow of air/fuel mixture during the suction stroke displays a turbulent and irregular flow. Also, this flow has the highest velocity and highest frequency among all component movements under the control of the VVT system^39^. Both of these characteristics match the I8 IMF, which has the highest frequency, at around 0.375 MHz, and is also turbulent and irregular, as can be seen in Fig. 2b. Hence, it is reasonable to assume this as evidence that I8 represents SG-noise. In the same context, 17, which has the second-highest frequency of 0.2439 MHz, as shown in Fig. 2b, has characteristics matching that of the bouncing movement of exhaust gases flowing out through the overlap process^36–38^. This can therefore be taken as an evidence that I7 is attributed to the OVT-noise. In “Cross-correlation of IMFs in different speeds” section, the cross-correlation technique will be used to further strengthen these evidences.

As was mentioned earlier, there are similarities in modes I5 and I6 as shown in Fig. 2b. It should be noted that both I5 and I6 have successive on–off functions during operation. The on–off sequence for I6 represents the intake valve closing time (IVCT) and intake valve opening time (IVOT) respectively. Also, the on–off sequences in I5 represent the exhaust valve closing time (EVCT) and exhaust valve opening time (EVOT) respectively. It worthily mentioning; that the frequency of I6 is around to 0.05 MHz while, I5 is 0.048 MHz.

The successive peaks in I5 and I6 (EVCT and IVCT) represent the sound wave energy due to the valves successively colliding with the engine body during the valve closing period. The regularity and uniformity of these peaks can be regarded as a signal indicating the safety of these valves.

As the movement of valves is controlled by camshafts, camshaft motions and their lobes were analyzed to explain the link between EVCT and IVCT from one side and I5 and I6 from the other.

**Figure 3:**
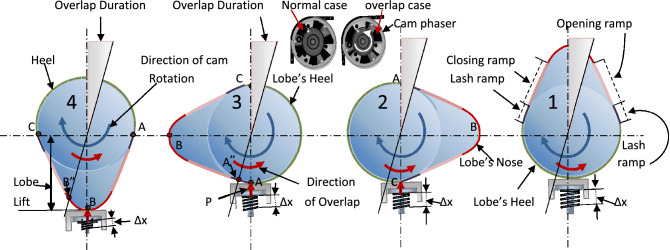
Cams profile of intake camshaft for Dodge Journey 2.4 engine according to firing order 1–3–4–2.

Figure 3 illustrates the location and motion of the intake camshaft lobes, describing the link between I6 and the lobe position. Point A in all lobes is the separation point between the opening and closing of the intake valve. The figure follows the the 1–3–4–2 cylinder firing order of the Dodge Journey engine^34^. When lobe-4 completes slipping over the follower and reaches point A, the valve hits the engine body. This moment is represented as one of the start points of IVCT in I5 as shown in Fig. 2b. During this rotation of lobe-4 from B to A, the valve opens and is depicted as one of the periods of IVOT. When the contact point between lobe-4 and its follower reaches point A, the contact point of lobe-2 reaches point B. Continuing further, when the contact point of lobe-2 reaches point A, the next IVCT starts, as shown in Fig. 2b.

Close examination of Fig. 2b, especially focusing on I5 and I6, reveals that the period length of A–A″ in I5 and I6 is twice that of the other periods. To explain this, we again refer to Fig. 3. Point B″, in lobe-4, returns to become the contact point instead of point B during the overlap period. The purpose of this overlap is to increase the opening time of the intake valves as the engine velocity increases from 650 to 1000 rpm. At the same time, Point A″ in lobe-3 will also return to act as the contact point with its follower at A. When this happens, the clash between the intake valves and engine body will repeat. This explains why the period of EVCT (A–A″) in I5 longer than the other EVCTs for I5. Consequently, it can be assumed that the extended period of A–A″ is contributed by the overlap period.

The group of IFMs from I1 to I4, in Fig. 2b represents the low frequencies whereas, were around (0.007–0.002) MHz. These IMFs have many shared characteristics such as (a) the shape of each IMF resembles a sinusoidal wave (b) modes on both sides of line Y–Y″ are similar form (c) for each high peak on the left-hand side of Y–Y″, there is a similar counterpart on the other side (see points E and E″ F and F″ G and G″ and H and H″), within the same IMF level (e) the level of energy, for all peaks, on the left-hand side is always higher than that on the right-hand side. These characteristics can be attributed to the frictional movements between both intake and exhaust camshafts and the pads located over the rocker arms.

The continuous friction between the camshaft and its rocker arm pad creates a continuous sound. It is known that a continuous sound resembles a sinusoidal waveform^40^. There is synchronization in the movements between both intake and exhaust camshafts because they are both controlled by the same engine control unit (ECU). In practice, the intake camshaft leads the exhaust camshaft by an angle called the separation angle. This is exactly what is illustrated in Fig. 2b, where the right-hand side, which represents the intake camshaft friction noise, leads the exhaust camshaft, represented on the left-hand side, in time. Practically, high temperature is the reason why the left-hand side has a higher energy level than the right-hand side. The increase in temperature in the exhaust camshaft causes increased extension in the mechanical moving parts and increases internal thrust. This thrust increases friction which in turn, increases the noise energy.

It should be noted that the noise due to friction between the camshafts and their pads has four important peaks at E, F, G, and H as shown in Fig. 2b. Referring to Fig. 3, the camshaft elliptical lobe cross-sectional profile also has four parts: lobe's nose, opening ramp, lash ramp, and lobe's heel. Both the energy and the sound mode type in this area (I1–I4 in Fig. 2b) depend on which part of the lobe profile is the contact point at that instance. As an example, it would be fair to consider that the high peak at point G represents the friction between the lobe's nose and its pad as the friction force at this point is at a maximum, $F_{f}$, which in turn, depends on the compressive force, $P$,where $F_{f} = \Delta x \times P$.

### Synchronization through the overlap

Figure 2b illustrates that there is a sequence of events (opening and closing) between intake valve closing time (IVCT) and exhaust valve closing time (EVCT). This is practically the same succession of opening and closing of both intake and exhaust valves in the dual VVT gasoline engine during idling^41^.

Overlap is the occurrence when both the intake and exhaust valves are open at the same time. Figure 2b confirms this synchronization at peak G, which occurs at the same time as the overlap in I5 (in region A–A″) on the right-hand side of line Y–Y″. A similar synchronization can be seen between G″ and A–A″ in I6, where G and G″ represent the lobe-4 position in Fig. 3 (opening of both intake and exhaust valves).

### Cross-correlation of IMFs in different speeds

In this study, the main goal of using the cross-correlation (Xcorr) is to prove the continuous similarity between I5 and I6 from the side and I7 and I8 from another side^41^, even though the gradual increase in speed from 1000 to 4000 rev/min.

**Figure 4:**
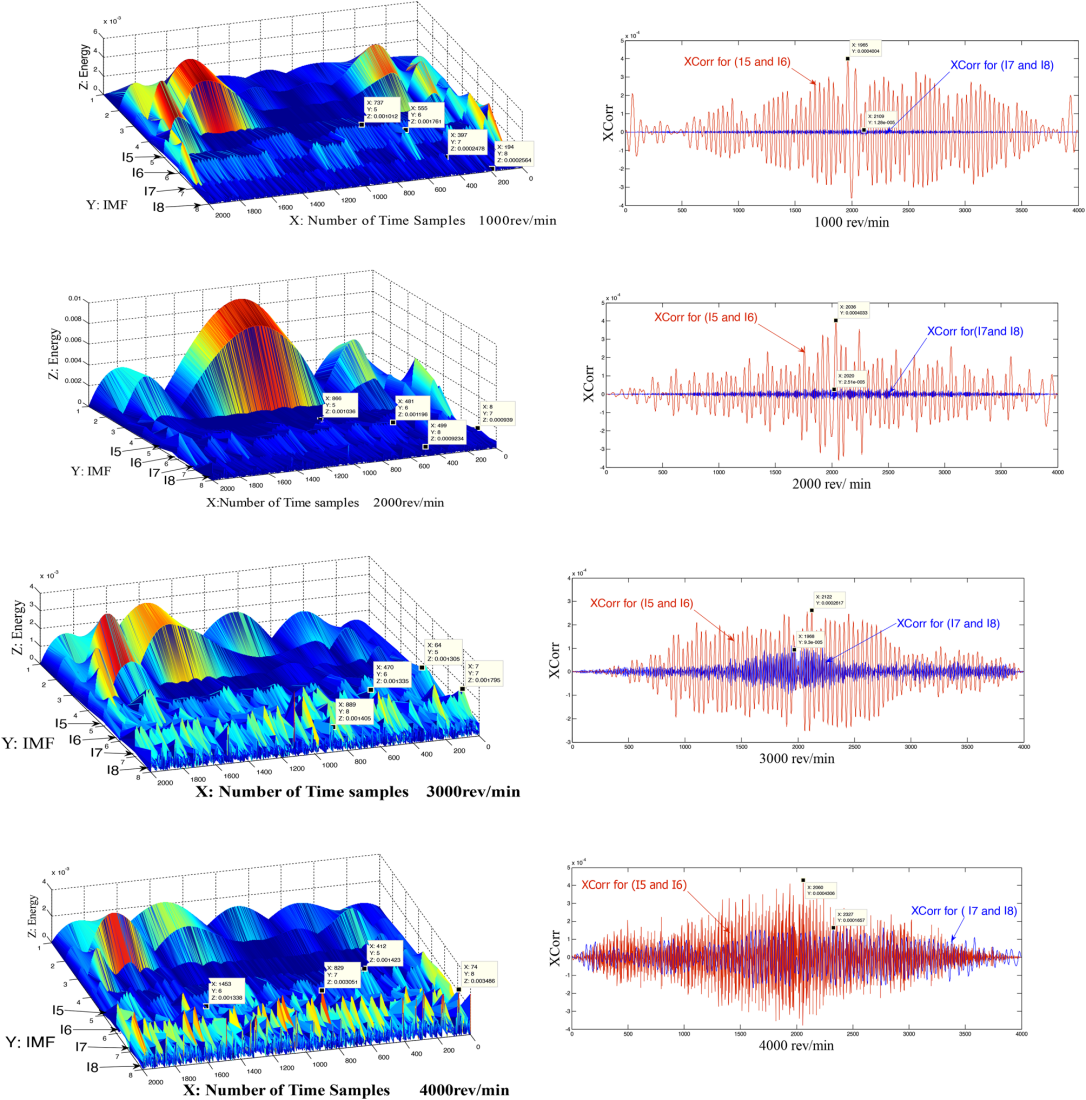
XCorr for I_8_ with I_7_ and I_5_ with I_6_ at different speeds.

One of the features of HHT is to analyze the signal to independent modes^31^. This feature indicates that each mode belongs to a specific mechanical part within the engine. Although I7 is independent of I8, Fig. 4 proves the persistent similarity between them (the blue color of Xcorr in Fig. 4) despite the speed increase from 1000 to 4000 rev/min. It is so clear that all figures of Fig. 4 are even-functions (symmetric about Y-axis), where the maximum peaks were at zero points for all cases of Xcorr for I7 and I8. The same thing happened between I5 and I6, which means that these two groups (I7–I8 and I5–I6) are subject to the same source. This source in these cases is the car's computer. It also means that each group (I7–I8 or I5–I6) has the same characteristics and in the case of I7 and I8 have the same path (from the combustion chamber to the meeting room as shown in Fig. 1).

On the other hand, Fig. 4 (energy time–frequency) proves that the level of sound of I5 and I6 stayed at 0.001, even though the increase of speed, while the level of I7 and I8 increased gradually, paralleling with increase the speed, from 0.0002 at 1000 rev/min to around 0.003 at 4000 rev/min. These results confirm the conclusions that were gone through this study about that the I5 and I6 were resulted due to the sound of hitting the valves to the engine body. The strike force level does not change with increasing the engine speed, but, the number of these hitting will increase with increasing engine speed. This explains why the level of I6 and I5 remained constant at the level of 0.001. While the level of I7 and I8 increased because I7 and I8 were resulted due to the suction processes of a mixture of air–fuel and that processes increase with increasing the engine speed. Also, the frequencies of I7 and I8 increased from around 0.3 to 0.8 MHz because of the increase in the flow of air–fuel mixture through increasing engine speed.

All these evidences were discussed in detail in this section. It can summarize, in brief, all that was reach above: (a) I8 represents the noise of air/fuel mixture suction (b) I7 is the noise of OVT-noise illustrated in Fig. 1c I5 and I6 represent the noise of both intake and exhaust valves respectively (d) finally from I1 to I4 are the noise due to friction between the camshaft and their rocker arm pads for both intake and exhaust camshafts. The points from (a to c) are enough to detect some of the engine faults. In addition, there is no room to discuss point (d) in the next section (“Detection of the combustion chambers damage through performance exam for engine dual camshaft” section) of this study.

It is worth mentioning; the microphone used couldn't detect the noise of the explosion and exhaust gas because the exhaust paths are designed in such a way that they reduce the sound to the minimal level so that it is hard to spot from the front of the car.

## Detection of the combustion chambers damage through performance exam for engine dual camshaft

**Figure 5:**
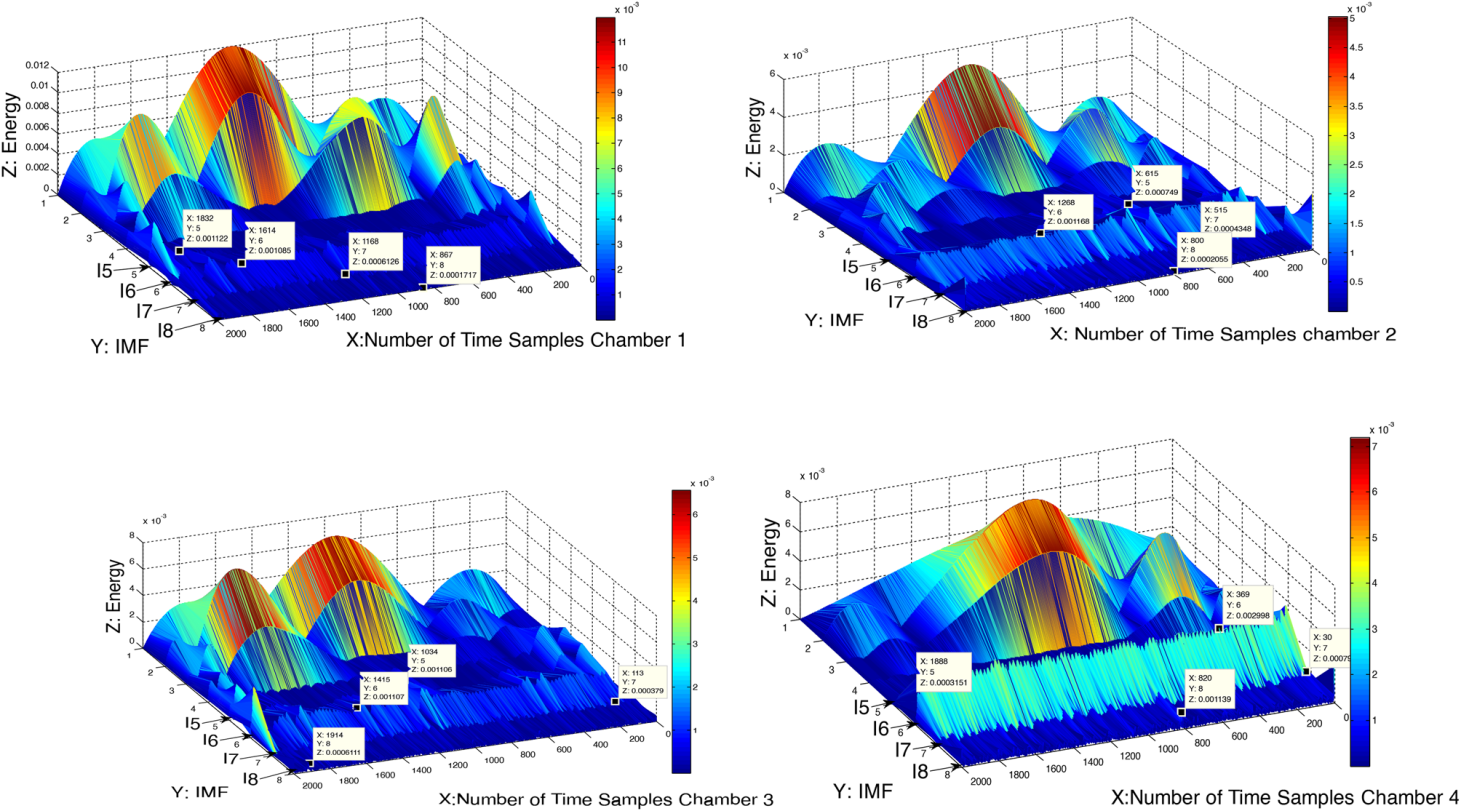
Illustrated the distribution of energy in time–frequency plan in cases of lifted the spark plug cable for the four chambers at 1000 rev/min.

First, in the practical part, the lifting of the cable that feeds a spark plug for the first combustion chamber was done, and then the engine sound, in this condition, was recorded. This process was repeated for chambers two, three then four. Figure 5 illustrated the distribution of energy time–frequency for the recorded data for those four cases were mentioned.

Figure 5 shows rising in the peaks number of I6, which represents the exhaust valve strokes of the engine body, while there is a lessening on the peaks of I5 due to the increase of the opening period intake valves.

Figure 5 revealed that the energy changes in both I6 and I8, in all four cases of Fig. 5, were significantly more than in I5 and I7. Therefore, this study focused on these two factors (I6 and I8) to find out the difference between these cases.

Practically, each of these four cases in Fig. 5 had a different tone of sound than the others. To revealing of this difference, the root-mean-square value (RMS) for the energy of sound, shown in Eq. (9), was used for each case individually for both I6 and I8. The symbol SN, in Eq. (9), represents the number of oscilloscope samples taken in each test in this study, where SN equals 2000 samples.

9 $$RMS = \sqrt {\frac{{\mathop \sum \nolimits_{j = 1}^{K} x^{2} (t_{j} )}}{SN}}$$

**Figure 6:**
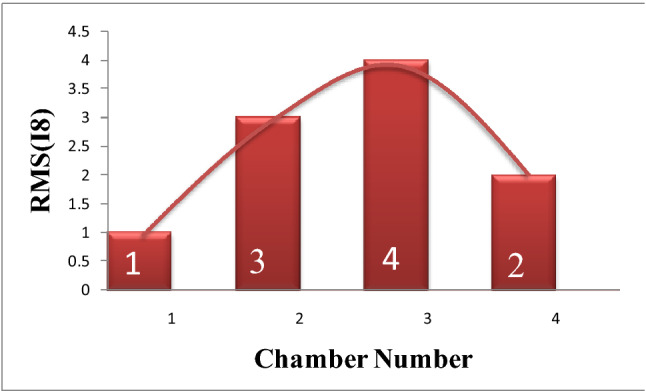
the value of I8 in each case for these four cases.

The results of using Eq. (9) are shown in Fig. 6. The arrangement of the cylinders (1–3–4–2) in Fig. 6 was according to the firing order (1–3–4–2) of the Dodge Journey. Here in this study, the values of RMS for any case represent the energy of engine sound waves in this case. This energy of sound waves indicates the activity of the mechanical parts of the engine in each case. It was observed, during those tests, at a speed of 650 rpm, there is a slight difference between the performances of combustion chambers (a slight difference in the sound power level of the engine). The role of the VVT system in compensation for any lack of engine power complicates the diagnosis of which of chambers has a problem, especially at low speed (650 rev/min). Therefore, these tests were done at 1000 rev/min to cut down on the ability of the VVT system to compensate for the lack of engine capability. Practically, at 1000 rev/min there were differences in the sounds of the engine in each test. That means the difference in I8 values will become greater for each case, where I8 refers to the condition of air/fuel charge entering the engine.

Practically, Fig. 6 illustrated the value of I8 for each case (test cases referenced). On the other hand, the set of.

Equations (10)–(11) clarifies the link between the chamber location that has a misfire and the value of negative torque of the engine for this case.

10 $$T_{Total} = T_{chemb} - T_{load} - T_{mass} - T_{fr}$$

where $T_{Total}$ is the total torque of the chamber, $T_{chemb}$ is the torque due to the combustion process, $T_{mass}$ is torque producing from reciprocating masses, $T_{load}$ is the load torque, and $T_{fr}$ represents the torque producing from the friction^42,43^. $T_{chemb} = 0$ at the moment that the electric plug is removed. On account of that,$T_{Total}$ becomes a negative ($- T_{Total}$). This means that $- T_{Total}$ workscontra the direction of rotation of the engine in this case. So the position of this negative torque becomes very poignant. Equation (11) indicates the effect of the position of the torque on the total torques at each end^44^.

$$\psi_{AC} = \psi_{BC} \quad \ldots where \;\psi \;is\;the\;twisting\;angle$$

$$\frac{{T_{B} L_{1} }}{{J_{BC} RS_{BC} }} = \frac{{T_{A} L_{2} }}{{J_{AC} RS_{AC} }}$$

$$T_{B} L_{1} = T_{A} L_{2} \quad \ldots where\;J_{AC} RS_{AC} = J_{BC} RS_{BC}$$

RS is the ratio between shear stress to shear strain and J is the moment of Inertia

11 $$T_{A} = \frac{{T_{B} L_{1} }}{{L_{2} }}$$

**Figure 7:**
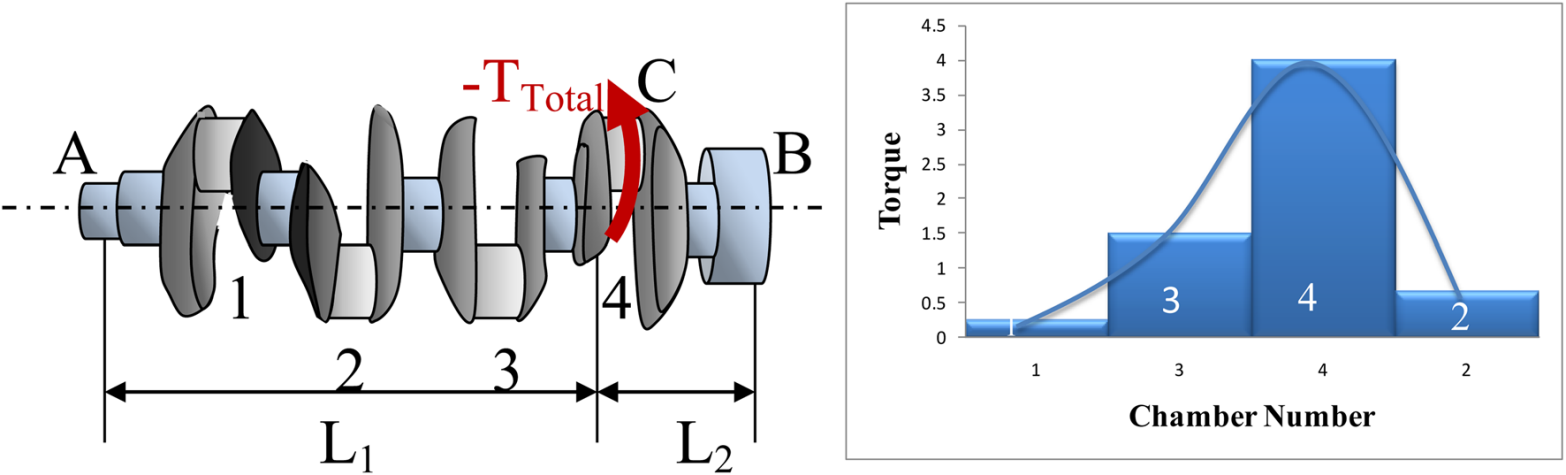
(**a**) Sketch of the effect of the position of $- T_{Total}$ and (**b**) column chart of Eq. (11) results for the torque at A.

The symbols of Eq. (11) was indicated in Fig. 7a

Figure 7b represents the results of this set of equations. The results illustrated that the highest value in both of these two figures (Figs. 6, 7b) is due to number chamber 4 then 3 then 2 and finally, chamber 1. Actually, in engine performance, there is a relation between noise level, fuel consumption, and generated torque. One of the roles of the VVT system is the increase the amounts of pumping the fuel to intact combustion chambers if there is a misfire in one of the combustion chambers^45^. Figures 6 and 7 prove, in idle case, that the VVT system pumps a large amount of fuel when chamber 4 has a problem because this chamber has the largest negative torque in this case. The same processes were conducted for chamber 3 then 2 then 1. Therefore, through conducting a comparison among the RMS values of I8 for each test, could be diagnosed which cylinder has a problem through the energy of sound.

## Conclusion

The significance of this research lies in diagnoses the sound of each mechanical part controlled by the VVT system, during its laboring when they are intact without any faults, through presenting the energy of engine sound within the time–frequency plane. This will help in the early prediction of the faults especially during the periodic maintenance of the car. The noise-based test was utilized to record the engine sound during the idle case. The high-resolution characteristic is one of the used oscilloscope features. This feature had a main role in the analysis of the engine sound. Also, the unidirectional pickup pattern had an important role in avoiding noise interference from other sources. The movements of five mechanical parts were diagnosed. The sound of movements of five mechanical parts was diagnosed. These parts act as a group inside the engine and are controlled by the VVT system. These sounds are the sound of the intake air/fuel mixture, overlap operation sound, the sound of hitting of both of intake and exhaust valves, and sound of slipping of both of intake and exhaust camshafts on rocker arm pad. The best speed for test the Dodge Journey engine car was 1000 rev/min because the capability of the VVT system will not be enough to compensate for the reduction in the engine power because of the misfire. These diagnoses were exploited in the detection of the faults of the engine combustion chambers. When the spark plug was removed for combustion chambers one by one at speeds of 650 rev/min and 1000 rev/min, the highest RMS of energy for noise was for combustion chamber number four. After removing the spark plug one by one during the mentioned tests, the sequence of the RMS of energy of engine sound, according to the arrangement of combustion chambers work (1–3-4–2), was equivalent and similar to the sequence of engine torque which calculated theoretically in these tests. As a result of this sequence, the target (the faulted combustion chamber) can be easily identified by using this sequence. It can say, any change occurs in the signal of any of these five mechanical parts, controlled by the VVT system, it can be early diagnosed.
