# Author Correction: Using energy time–frequency of Hilbert Huang transform to analyze the performance of the variable valve timing engine

**DOI:** 10.1038/s41598-022-11360-z

**Published:** 2022-05-02

**Authors:** Arshed Abdulhamed Mohammed, Sallehuddin Mohamed Haris

**Affiliations:** 1 Department of Materials Engineering College of Engineering, University of Diyala, Baquba, Diyala Iraq; 2 Department of Mechanical and Manufacturing Engineering, Faculty of Engineering and the Built Environment, Universiti Kebangsaan Malaysia, 43600 UKM, Bangi, Selangor Malaysia

Correction to: *Scientific Reports* https://doi.org/10.1038/s41598-022-06404-3, published online 11 February 2022

The original version of this Article contained errors in the Reference list, where references 1,2,4,5,12,13,14,15,18,21,22,23,34,36,37,39,40,41,42,43,44 and 45 were incorrectly given as

1. Rosero, J., Garcia, A., Cusido, J., Romeral, L. & Ortega, J. A. in *IEEE International Symposium on Intelligent Signal Processing, 2007. WISP 2007*. 1–6.
2. Yang, Z., Yu, Z., Xie, C. & Huang, Y. Application of Hilbert-Huang Transform to acoustic emission signal for burn feature extraction in surface grinding process. *Measurement* **47**, 14–21. https://doi.org/10.1016/j.measurement.2013.08.036 (2014).
3. Ryu, J., Carazo, A., Uchino, K. & Kim, H.-E. Piezoelectric and magnetoelectric properties of lead zirconate titanate/Ni-ferrite particulate composites. *J. Electroceram*. **7**, 17–24. https://doi.org/10.1023/A:1012210609895 (2001).
4. Lach, M., Platte, M. & Ries, A. Piezoelectric materials for ultrasonic probes. T*he Open Access NDT Database* **9** (1996).
5. Fukada, E. & Yasuda, I. On the piezoelectricity of bone. *Jpn. J. Appl. Phys*. **3**, 117–121 (1964).
6. Kawai, H. Te piezoelectricity of poly(vinylidene fluoride). *Jpn. J. Appl. Phys*. **8**, 975–976 (1969).
7. Wear, K. A., Gammell, P. M., Maruvada, S., Liu, Y. & Harris, G. R. Improved measurement of acoustic output using complex deconvolution of hydrophone sensitivity. *IEEE Trans. Ultrason. Ferroelectr. Freq. Control* **61**, 62–75. https://doi.org/10.1109/TUFFC.2014.6689776 (2014).
8. Zuo, X. et al. Desalination of water with a high degree of mineralization using SiO2/PVDF membranes. *Desalination* **311**, 150–155.
9. Panwar, V., Ko, S. Y., Park, J.-O. & Park, S. Enhanced and fast actuation of fullerenol/PVDF/PVP/PSSA based ionic polymer metal composite actuators. *Sens. Actuators B Chem*. **183**, 504–517. https://doi.org/10.1016/j.snb.2013.04.037 (2013).
10. Stuetzer, O. M. Multiple refections in a free piezoelectric plate. *J. Acoust. Soc. Am.* **42**, 502–508. https://doi.org/10.1121/1.1910607 (1967).
11. Day, R. A. PVDF and array transducers. *The Open Access NDT Database* **9** (1996).
12. Lei, X.-R. et al. Effect of niobium on dynamic recrystallization behavior of 5% Ni steel. *J. Iron Steel Res Int.* **20**, 38–44. https://doi.org/10.1016/S1006-706X(13)60109-0 (2013).
13. Cheng, G., Cheng, Y.-L., Shen, L.-H., Qiu, J.-B. & Zhang, S. Gear fault identification based on Hilbert-Huang transform and SOM neural network. *Measurement* **46**, 1137–1146. https://doi.org/10.1016/j.measurement.2012.10.026 (2013).
14. Cheng, J., Yu, D., Tang, J. & Yang, Y. Application of frequency family separation method based upon EMD and local Hilbert energy spectrum method to gear fault diagnosis. *Mech. Mach. Theory* **43**, 712–723. https://doi.org/10.1016/j.mechmachtheory.2007.05.007 (2008).
15. Zhu, D., Gao, Q., Sun, D., Lu, Y. & Peng, S. A detection method for bearing faults using null space pursuit and S transform. *Signal Process*. **96**(Part A), 80–89. https://doi.org/10.1016/j.sigpro.2013.04.019 (2014).
16. Pines, D. & Salvino, L. Structural health monitoring using empirical mode decomposition and the Hilbert phase. *J. Sound Vib*. **294**, 97–124. https://doi.org/10.1016/j.jsv.2005.10.024 (2006).
17. Hamdi, S. E. et al. Acoustic emission pattern recognition approach based on Hilbert-Huang transform for structural health monitoring in polymer-composite materials. *Appl. Acoust.* **74**, 746–757. https://doi.org/10.1016/j.apacoust.2012.11.018 (2013).
18. Redwood, M. Transient performance of a piezoelectric transducer. *J. Acoust. Soc. Am.* **33**, 527–536. https://doi.org/10.1121/1.1908709 (1961).
19. Wang, Y. S., Ma, Q. H., Zhu, Q., Liu, X. T. & Zhao, L. H. An intelligent approach for engine fault diagnosis based on Hilbert-Huang transform and support vector machine. *Appl. Acoust*. **75**, 1–9. https://doi.org/10.1016/j.apacoust.2013.07.001 (2014).
20. Dybała, J. & Zimroz, R. Rolling bearing diagnosing method based on Empirical Mode Decomposition of machine vibration signal. *Appl. Acoust.* **77**, 195–203. https://doi.org/10.1016/j.apacoust.2013.09.001 (2014).
21. Kalvoda, T. & Hwang, Y.-R. Analysis of signals for monitoring of nonlinear and non-stationary machining processes. *Sens. Actuators A Phys*. **161**, 39–45. https://doi.org/10.1016/j.sna.2010.05.032 (2010).
22. Cao, H., Lei, Y. & He, Z. Chatter identification in end milling process using wavelet packets and Hilbert-Huang transform. *Int. J. Mach. Tools Manuf.* **69**, 11–19. https://doi.org/10.1016/j.ijmachtools.2013.02.007 (2013).

The correct references are the following:

1. Renshaw, J. Variable Valve Timing (VVT) Systems. *Advice & How-Tos / Car Technology* (11–9-2019).
2. Markel, A. Diagnosing Variable Valve Timing. *Brake and Front End *https://www.brakeandfrontend.com/diagnosing-variable-valve-timing/ (Mar 4, 2014).
3. Abdullah, S., Deros, B. M., Khamis, N. K., Ghani, J. A. & Abdullah, S. An approach to work Design: in-depth: audit to determine the modifiers of musculoskeletal disorder symptom among vehicle maintenance personnel. *Jurnal Kejuruteraan* **2**, 281–287 (2020).
4. Abdullaha, S., Khamisa, N. K., Ghania, J. A. & Kurniawanb, R. Posture Evaluation of the Automotive Maintenance Workers: A Case Study. *Jurnal Kejuruteraan* **3**, 65–70 (2020).
5. Ahmed, R., Sayed, M. E., Gadsden, S. A., Tjong, J. & Habibi, S. Automotive internal combustion engine fault detection and classification using artificial neural network techniques. *IEEE Trans. Veh. Technol.* **64**, 21–33 (2014).
6. Shah, M., Gaikwad, V., Lokhande, S. & Borhade, S. Fault identification for I.C. engines using artificial neural network. *International Conference on Process Automation, Control and Computing (PACC)* (2011).
7. Chen, J., Patton, R. J. & Liu, G.-P. Optimal residual design for fault diagnosis using multi-objective optimization and genetic algorithms. *Int. J. Syst. Sci.* **27**, 567–576 (2015).
8. Bay, Ö. F. & Bayir, R. R. A fault diagnosis of engine starting system via starter motors using fuzzy logic algorithm. *Gazi Univ. J. Sci.* **24**, 437–449 (2011).
9. Wu, J.-D. & Chen, J.-C. Continuous wavelet transform technique for fault signal diagnosis of internal combustion engines. *NDT&E Int.* **39**, 304–311 (2006).
10. Candon, M. et al*.* Characterization of a 3DOF aeroelastic system with freeplay and aerodynamic nonlinearities – Part II: Hilbert–Huang transform. *Mech. Syst. Signal Process.* **114**, 628–643 (2019).
11. Nasir, N. N. M. et al*.* Time–frequency strain data analysis of suspension using the Hilbert-Huang transform. *J. Mech. Eng.* **1**, 59–77 (2018).
12. Nasir, N. N. M., Singh, S., Abdullah, S. & Haris, S. M. Accelerating the fatigue analysis based on strain signal using Hilbert–Huang transform. *Int. J. Struct. Integrity* **10**(1), 118–132(2019).
13. DODGE-Journey-Forum.com. https://www.dodgejourneyforum.com/topic/6371-cutting-out/ (2020).
14. Sawant, P. & Bari, S. combined effects of variable intake manifold length, variable valve timing and duration on the performance of an internal combustion engine. In *Proceedings of the ASME 2017 International Mechanical Engineering Congress and Exposition IMECE2017* (2017).
15. Bell, A. G. *Four-Stroke Performance Tuning* (Cambridge University Press, 1998).
16. Torenbeek, E. & Wittenberg, H. Low-speed aerodynamics, in *Flight Physics* (Springer, 2009).
17. Picard, C. e., Tsingos, N. & Faure, F. Retargeting Example Sounds to Interactive Physics-Driven Animations. In *AES 35th International Conference* (2009).
18. Hadfield, C. & Nussler, R. *Automotive Engine Repair & Rebuilding, Classroom Manual and Shop Manual* (Spiral bound Version. Cengage Learning, 2017).
19. Nayeri, M. H. Cylinder-by-Cylinder Torque Model of an SI-Engine for Real-Time Applications, Master thesis (Avdelning Institution, 2005).
20. Kiencke, U. & Nielsen, L. *Automotive Control Systems for Engine, Driveline, and Vehicle* (Springer, 2005).
21. Hibbeler, R. C. *Mechanics of Materials* (Prentice Hall, 2014).
22. Park, D., *System for variably controlling operation of an intake/exhaust valve for an internal combustion engine*, in *United States Patent* (Hyundai Motor Company, Seoul, 1999).

The original Article has been corrected.

